# Inequality in benefit distribution of reducing the outpatient cost-sharing: evidence from the outpatient pooling scheme in China

**DOI:** 10.3389/fpubh.2024.1357114

**Published:** 2024-03-04

**Authors:** Tao Zhang, Minyan Chen

**Affiliations:** ^1^Department of Health Policy and Management, School of Public Health, Hangzhou Normal University, Zhejiang, China; ^2^Medical Insurance Department, Hangzhou Ninth People’s Hospital, Zhejiang, China

**Keywords:** cost-sharing, benefits equity, healthcare needs, concentration index, China

## Abstract

**Objective:**

The implementation of the outpatient pooling scheme in China has substantially elevated the compensation levels for outpatient expenses. This study aims to assess whether socioeconomically disadvantaged enrollees benefit proportionally compared to their non-disadvantaged counterparts.

**Method:**

A cohort comprising 14,581 Urban and Rural Resident Basic Medical Insurance (URRBMI) enrollees and 830 Urban Employee Basic Medical Insurance (UEBMI) enrollees was derived from the China Health and Retirement Longitudinal Study 2018. Outpatient pooling scheme benefits were evaluated based on two metrics: the probability of obtaining benefits and the magnitude of benefits (reimbursement amounts and ratios). Two-part models were employed to adjust outpatient benefits for healthcare needs. Inequality in benefit distribution was assessed using the concentration curve and concentration index (CI).

**Results:**

Following adjustments for healthcare needs, the CI for the probability of receiving outpatient benefits for URRBMI and UEBMI enrollees were − 0.0760 and − 0.0514, respectively, indicating an evident pro-poor pattern under the outpatient pooling scheme. However, the CIs of reimbursement amounts (0.0708) and ratio (0.0761) for URRBMI recipients were positive, signifying a discernible pro-rich inequality in the degree of benefits. Conversely, socioeconomically disadvantaged UEBMI enrollees received higher reimbursement amounts and ratios.

**Conclusion:**

Despite a higher likelihood of socioeconomically disadvantaged groups receiving outpatient benefits, a pro-rich inequality persists in the degree of benefits under the outpatient pooling scheme in China. Comprehensive strategies, including expanding outpatient financial benefits, adopting distinct reimbursement standards, and enhancing the accessibility of outpatient care, need to be implemented to achieve equity in benefits distribution.

## 1 Introduction

Ensuring health equity is recognized as a fundamental human right. Inequality in access to healthcare is considered politically unacceptable and unjust. However, the widening disparities in income or socioeconomic status (SES) have led to persistent inequalities in health and access to healthcare services (1). To mitigate health inequality, various health reforms aimed at achieving Universal Health Coverage (UHC) have been implemented in several countries (2, 3). In the practice of UHC reform, government-subsidized health insurance schemes have been viewed as levers to facilitate a pro-poor distribution of public resources and improve health equity, although taxation also plays a significant role in many countries (4). A Study conducted in India found social health insurance played a significant role in reducing financial hardship (5). Additionally, a systematic review also suggests that micro health insurance, targeted at the low-income households, may contribute toward providing protection to the households from catastrophe and impoverishment (6).

Since the 1990s, China has been striving to establish a new Social Health Insurance system (SHI). By 2010, over 95% of the Chinese population was covered by three major health insurance schemes: Urban Employee Basic Medical Insurance (UEBMI) (7), Urban Resident Basic Medical Insurance (URBMI), and the New Cooperative Medical Scheme (NCMS). In 2016, URBMI and NCMS were integrated into the Urban and Rural Resident Basic Medical Insurance (URRBMI) (8).The UEBMI is China’s largest social medical insurance plan in terms of fund revenue and surplus. It is primarily financed by payroll taxes paid by both employers (6%) and employees (2%) on a monthly basis. Premiums for URRBMI were jointly paid by households and the government on a yearly basis. Data showed that the annual contribution *per capita* for UEBMI and URRBMI in 2020 was 4,566 yuan and 896 yuan, respectively (9). All enrollees can get immediate reimbursements after using services covered by the insurance. Patients do not need to apply for retrospective reimbursement. In other words, patients only need to pay out-of-pocket payments at the outpatient delivery point. Nonetheless, information on total expenses and out-of-pocket (OOP) payments is provided for each outpatient visit. In terms of financial benefits, both UEBMI and URRBMI emphasize cost-sharing for enrollees and introduce complex rules regarding deductibles, copayments, and maximum reimbursements, but the financial benefits of UEBMI are higher than those of URRBMI (9). Details for UEBMI and URRBMI are available in Supplementary Table S1.

As a means of risk-sharing, China’s social health insurance system is designed to eliminate financial barriers to accessing health services, especially for vulnerable groups. All enrollees receive an equal amount of premium subsidy from the government and enjoy the same financial benefits (10, 11). Although the equal policy design appears to guarantee equal opportunities for every enrollee to enjoy its benefits, it does not necessarily result in equitable benefit distribution. Some early studies have indicated that better-off individuals benefit more from Chinese SHI because they utilize more healthcare services than socioeconomically disadvantaged groups (11, 12).

Among several contributors associated with the non-equitable distribution of benefits in China’s basic medical insurance system (e.g., individual income, health status, and education), inadequate financial support for outpatient services should not be overlooked. In the initial stages of establishing Chinese SHI, the financial benefits prioritized inpatient care but neglected compensation for outpatient expenses (13, 14). High out-of-pocket (OOP) payments for outpatient care hinder access to healthcare for low-income groups, resulting in fewer opportunities for the poor to obtain benefits than for high-income groups. Moreover, limited access to outpatient services for low-income groups may lead to delays in disease treatment and the development of more serious diseases, exacerbating health disparities among different income groups (15, 16).

In 2007, the Chinese central government proposed the establishment of outpatient pooling schemes in regions where conditions allowed. The core of this initiative is to establish an outpatient pooling fund and increase financial support for outpatient care. In response to this call, the URBMI/NCMS established an outpatient pooling scheme from 2009 to 2011. However, UEBMI only introduced outpatient pooling schemes in a few prefectures (such as Beijing and Shanghai) gradually since 2011, without nationwide implementation. In April 2021, China issued guidelines on the establishment of a mutual assistance security mechanism for outpatient care under UEBMI, aiming to explore a pooling mechanism for the costs of outpatient services (17). Overall, China is making significant efforts to reduce financial hardships and expand financial benefits for outpatient care. Therefore, against the backdrop of improving insurance generosity, understanding the distribution of insurance benefits becomes critically important.

Previous studies have provided evidence on the benefit distribution of medical insurance among insured populations across different economic levels. For example, Lai et al. (12) evaluated the distribution of benefits under NCMS across economic groups and found that benefits were concentrated toward economically affluent groups (12). Pan et al. (11) also found that lower-income groups benefited less than higher-income groups in China’s URBMI program (11). Liu and Dai (2020) observed that compared to the highest-income group, below-middle-income groups had significantly insufficient compensation in China’s basic medical insurance system (18). However, some gaps remain in existing studies. First, these evaluations mainly focused on the distribution of inpatient benefits, with limited analysis on outpatient benefits (19). Second, previous studies were conducted in the context of relatively low compensation levels for medical expenses, which may not fully apply to current situations due to improvements in insurance generosity in China in recent years, such as higher reimbursement ratios and an expansion of the scope of reimbursement. Third, published literature tends to assess income-related inequalities in insurance benefits, with little exploration of benefits distribution across other socioeconomic variables (e.g., education, employment) (20).

Focusing on outpatient benefits, this study aims to assess whether and to what extent the benefits distribution of outpatient pooling schemes differs across socioeconomic groups against the backdrop of a significant increase in the generosity of China’s basic medical insurance. Our findings provide meaningful policy implications for further reform of the outpatient pooling scheme in China and valuable lessons for improving the equity of health insurance benefits in other countries facing similar challenges.

## 2 Materials and methods

### 2.1 Data sources

The data utilized in this study were acquired from the China Health and Retirement Longitudinal Study (CHARLS), a comprehensive initiative that captures a nationally representative sample of individuals aged 45 and above in China. This longitudinal study provides individual-level panel data on health, socio-economic status (SES), and social and family networks at intervals of two to 3 years (21). The samples were meticulously selected using a multistage probability sampling approach. In the initial stage of sampling, county-level units were randomly selected utilizing a probability-proportional-to-size (PPS) sampling technique from a sampling frame encompassing all county-level units, excluding Tibet. The sampling process was stratified by region, and within each region, further stratification was carried out based on distinctions between urban districts and rural counties, along with *per capita* statistics on gross domestic product (GDP). The ultimate sample consisted of 150 counties distributed across 28 provinces (21). Within the CHARLS framework, questions specific to health insurance, including URRBMI (NCMS/URBMI) and UEBMI, were used to measure reimbursements insured person obtained. Thus, CHARLS stands out as a unique and valuable source for examining the equity of benefits provided by health insurance. Further details regarding the sampling process and data collection methods in CHARLS can be explored in other publications (21).

To date, a total of four waves of CHARLS (2011, 2013, 2015 and 2018) have been completed. Based on the following reasons, we adopted data from the latest survey conducted in 2018. Firstly, the ongoing reform of merging NCMS and URBMI into URRBMI was conducting from 2012 to 2016. Previous studies proved integrated URRBMI improves healthcare and benefit equity (8, 22). To eliminate the confounding effects of this policy, the data collected after 2016 is appropriate. Secondly, some cities in China started to pilot outpatient pooling scheme in UEBMI since 2001, but it was not implemented nationwide. Using the 2018 data allow us to obtain more UEBMI enrollees form prefectures implementing the outpatient pooling scheme.

In line with the study’s objective, which focuses on investigating the distribution of benefits in the outpatient pooling scheme within China’s basic health insurance system, respondents not covered by URRBMI and UEBMI were excluded from the analysis. Furthermore, it is essential to note that while the outpatient pooling scheme has been implemented nationwide for URRBMI, its introduction into UEBMI has been limited to only a few prefectures in China. Therefore, UEBMI enrollees from prefectures where the outpatient pooling scheme was not implemented were excluded from the study. Supplementary Table S2 (found in Supplementary Table S1) categorizes prefectures based on the implementation status of the outpatient pooling scheme within UEBMI in 2018. Ultimately, a total of 15,411 individual-level observations were available for data analysis, comprising 14,581 URRBMI enrollees and 830 UEBMI enrollees.

### 2.2 Variables

We divided the variables used in the research into three categories: (1) benefits obtained from outpatient pooling scheme; (2) individual socioeconomic status (SES); (3) healthcare need variables. The detailed definitions and measurements of variables can be found in Supplementary Table S3.

### 2.3 Benefits from the outpatient pooling scheme

We examined two types of benefit outcomes: (1) The probability of obtaining benefits, measured by the probability of URRBMI/UEBMI enrollees being reimbursed for outpatient expenses in the previous month (yes = 1; no = 0) (8); (2) the degree of benefits, which was measured by the total amount of reimbursements received and the reimbursement ratio among those who had received reimbursements for outpatient expenses in the previous month (11).

### 2.4 Socioeconomic status

Following the previous studies (23–25), the present study used four variables to assess individual SES, including residency (1 = urban, 2 = rural), educational level (1 = illiteracy, 2 = primary school, 3 = middle school, 4 = college or above), employment status (employed or unemployed) and annul income (1 = ‘<3,000 yuan’, 2 = ‘3,000–6,000 yuan’, 3 = ‘6,001–10,000 yuan’, 4 = ‘>10,000 yuan’).

### 2.5 Healthcare need variables

Considering the substantial influence of healthcare need factors on the distribution of benefits across various socioeconomic status (SES) groups, demographic characteristics (gender, age, and marital status) as well as health status indicators (self-rated health, chronic disease status, physical disabilities, and body pain) were employed to comprehensively reflect individual healthcare needs and to meticulously adjust the obtained benefits (12, 26).

Gender was dichotomized into male and female categories. Age was stratified into four groups: 45–55, 56–65, and 65 or above. Marital status was classified into two categories: married and other. Self-rated health was segmented into five distinct categories ranging from very poor to very good. Chronic disease status and physical disabilities were binary variables, defined as either present or absent. Body pain was categorized into three levels: none, a little, and quite. These variables collectively enable a nuanced understanding of individual healthcare needs and facilitate a robust adjustment of the benefits acquired in the analysis (13, 27).

### 2.6 Statistical analyses

This study investigated the benefits equity of outpatient pooling scheme, following the three steps. In the first step, the distribution of the probability of receiving reimbursements, reimbursement amount and reimbursement ratio were compared by residency, educational level, employment status and income using chi-square and non-parametric tests.

In the second step, following the “equal treatment for equal needs” concept, benefits should be determined based solely on healthcare, and not influenced by any other socioeconomic factors (27). Therefore, the confounding effects of healthcare needs on the benefits distribution should be adjusted (26). In this study, two-part models were used to eliminate confounding effects because the variables “reimbursements amount” and “reimbursement ratio” had a significant number of zero observations (28). In the first part, a logit model with the probability of obtaining benefits as the dependent variable and healthcare need variables as the independent variables was adopted. In the second part, the degree of benefits (reimbursement amounts and ratio), conditional on a positive probability of obtaining benefits, was then estimated using GLM (generalized linear model). Since the distribution of reimbursement amounts was highly skewed, the natural logarithm was used in the model (12).

In the third step, using the predicted probability of receiving reimbursement, reimbursement amounts and ratios based on the two-part models, the benefits equity was examined using concentration curve and concentration index (CI). The concentration curve depicts the cumulative proportion of the population, ranked by socioeconomic level from lowest to highest on the horizontal axis, against the cumulative proportion of benefits on the vertical axis (29). The 45-degree line represents perfect equality in benefits distribution. If the curve is above the 45-degree line, it indicates that benefits are more highly concentrated among the poor, and vice versa (29).

The CI is defined as twice the area between the concentration curve and the 45-degree line. The value of CI ranges between −1 and 1 with a negative value indicating that individuals with a lower SES benefit more from the outpatient pooling scheme and vice versa (30). The CI formula is as follows:


$$C=\frac{2}{\mu }COV\left(y,\gamma \right)$$

Where y denotes benefit outcomes and μ is the mean of y. the parameter γ represents a fractional rank in the socioeconomic distribution.

In our analysis, Principal Components Analysis (PCA) was performed to create a synthetic SES index using four indicators: residency, educational level, employment status and individual income (23, 31). PCA model results can be found in Supplementary Tables S4, S5. After PCA estimation, each individual-level observation was assigned an index value. A large value represents a high level of SES. Based on the index value, the sample was divided into four equally sized groups representing socioeconomic levels, ranging from lowest (Quartile 1, Q1) to highest (Quartile 4, Q4).

Due to the great differences in financial benefits and cost-sharing arrangements between URRBMI and UEBMI in China, data analysis was conducted separately for these two types of insurance enrollees. All analyses were performed using Stata 16.0 for Windows and 5% was set as the significance level.

## 3 Results

### 3.1 Description of respondents

Table 1 presents a comprehensive overview of the benefit outcomes and demographic as well as socio-economic characteristics of URRBMI and UEBMI enrollees.

**Table 1:** Characteristics of URRBMI and UEBMI enrollees.

Variables		URRBMI enrollees (*n* = 14,581)		UEBMI enrollees (*n* = 830)	
		n/mean	%/SD	n/mean	%/SD
Probability of getting reimbursed	Yes	341	2.3	142	17.1
	No	14,240	97.7	688	82.9
Reimbursement received (yuan)		1087.2	2798.7	1813.1	2965.9
Reimbursement ratio (%)		50.4	28.8	60.2	29.8
Gender	Male	6,632	45.5	447	53.9
	Female	7,949	54.5	383	46.1
Age (year)	45–55	5,067	34.8	288	34.7
	56–65	4,784	32.8	265	31.9
	>65	4,730	32.4	277	33.7
Marital status	Married	12,613	86.5	742	89.4
	Others	1968	13.5	88	10.6
Self-rated health	Very poor	915	6.3	23	2.8
	Poor	3,141	21.5	77	9.3
	Fair	7,061	48.4	432	52.0
	Good	1711	11.7	154	18.6
	Very good	1753	12.0	144	17.3
Chronic disease status	Yes	6,354	43.6	389	46.9
	No	8,227	56.4	441	53.1
Physical disabilities	Yes	399	2.7	13	1.6
	No	14,182	97.3	817	98.4
Body pain	None	5,476	37.6	436	52.6
	A little	6,239	42.8	325	39.2
	Quite	2,863	19.6	69	8.3
Residency	Urban	2,755	18.9	576	69.3
	Rural	11,825	81.1	255	30.7
Educational level	Illiteracy	3,483	23.9	30	3.6
	Primary school	6,941	47.6	195	23.5
	Middle school	4,120	28.2	511	61.6
	College or above	37	0.3	94	11.3
Employment status	Employed	10,148	69.6	378	45.5
	Unemployed	4,433	30.4	452	54.5
Annual income (yuan)	<3,000	10,241	70.2	73	8.8
	3,000–6,000	641	4.3	89	10.7
	6,001–10,000	572	3.9	114	13.7
	>10,000	3,127	21.4	554	66.7

The analysis indicates that UEBMI enrollees derived greater benefits from the outpatient pooling scheme compared to their URRBMI counterparts. For instance, the probability of UEBMI enrollees receiving reimbursements stood at 17.1%, significantly higher than the mere 2.3% probability observed among URRBMI enrollees. Furthermore, the average reimbursement amounts and ratios for UEBMI recipients were notably higher, at 1167.67 yuan and 51.91%, respectively, compared to those for URRBMI recipients.

On the demographic front, both URRBMI and UEBMI samples exhibited similar characteristics in terms of gender, age distribution, and marital status. In terms of health status, a notable proportion of both URRBMI and UEBMI enrollees reported very good health ratings (12.0 and 17.3%, respectively). Additionally, over 40% of respondents from both insurance schemes reported being diagnosed with chronic diseases, while approximately 40% experienced some degree of physical pain.

When examining socio-economic status (SES), UEBMI enrollees displayed a more advantaged profile compared to their URRBMI counterparts. For instance, a higher proportion of UEBMI enrollees had completed college education or above, reported an annual income exceeding 10,000 yuan, and resided in urban areas, in contrast to URRBMI enrollees.

### 3.2 Benefits distribution

**Figure 1:**
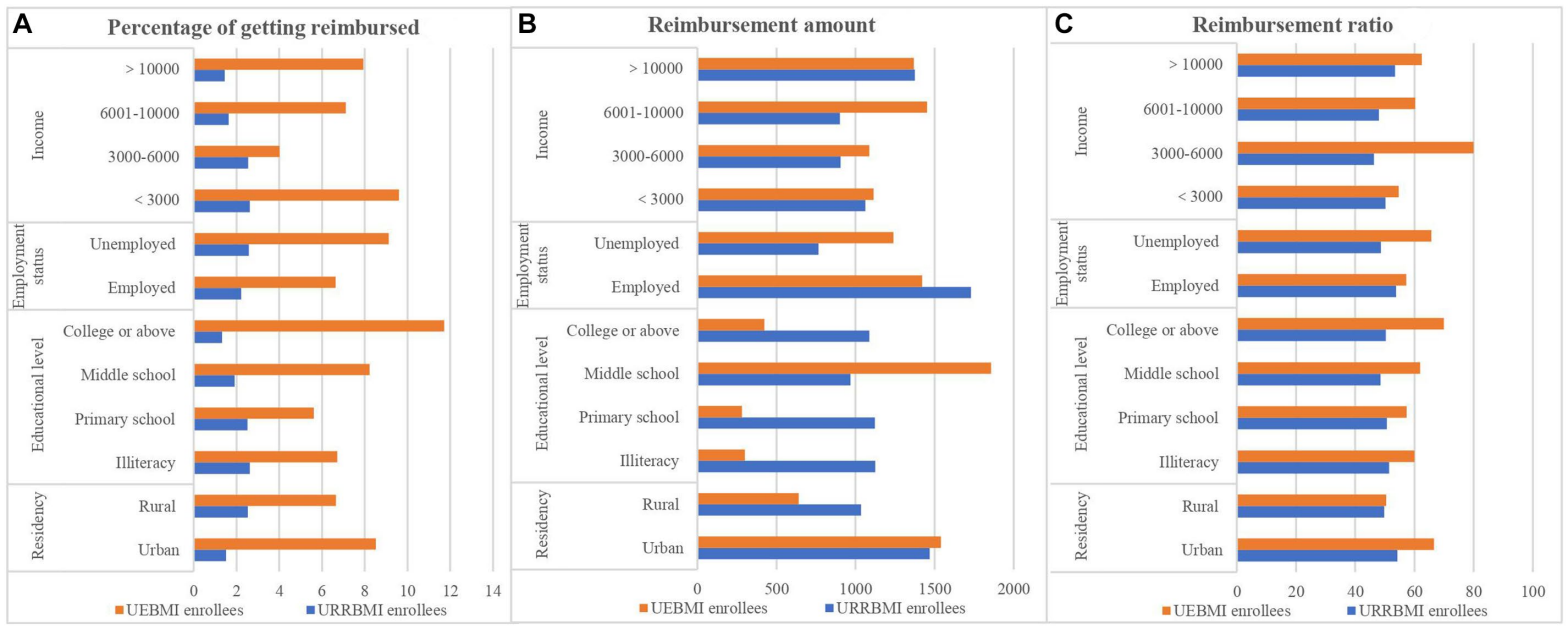
Distribution of percentage of getting reimbursed **(A)**, reimbursement amount **(B)** and reimbursement ratio **(C)** by SES groups.

The distribution of outpatient benefits from the pooling scheme exhibited variations across residency, educational level, employment status, and income groups, as elucidated in Table 2 and Figure 1. Overall, UEBMI enrollees demonstrated a higher receipt of outpatient benefits compared to URRBMI enrollees.

**Table 2:** Distribution of benefits by different SES groups.

Population groupings	URRBMI enrollees			UEBMI enrollees		
	Percentage of getting reimbursed (%)	Reimbursement amount (yuan)	Reimbursement ratio (%)	Percentage of getting reimbursed (%)	Reimbursement amount (yuan)	Reimbursement ratio (%)
By residency						
Urban	1.52	1468.45	54.29	8.52	1539.29	66.65
Rural	2.53	1033.62	49.83	6.66	639.59	50.41
value of p	0.002	0.347	0.348	0.362	0.207	0.012
By educational level						
Illiteracy	2.63	1124.80	51.44	6.71	300.00	60.00
Primary school	2.51	1123.10	50.69	5.62	281.82	57.33
Middle school	1.92	968.09	48.53	8.23	1855.88	61.97
College or above	1.33	1087.18	50.38	11.71	422.82	69.92
value of p	0.205	0.910	0.792	0.041	0.138	0.647
By employment status						
Employed	2.23	1728.75	53.76	6.63	1420.12	57.21
Unemployed	2.58	764.98	48.68	9.12	1238.90	65.67
value of p	0.196	0.003	0.124	0.193	0.779	0.155
By Income (yuan)						
<3,000	2.62	1060.99	50.26	9.60	1114.00	54.66
3,000–6,000	2.55	904.78	46.30	4.01	1086.36	80.00
6,001–10,000	1.63	901.82	48.03	7.11	1452.00	60.22
>10,000	1.45	1374.77	53.45	7.93	1368.60	62.46
value of p	0.002	0.897	0.820	0.846	0.934	0.272

Specifically, within the URRBMI cohort, rural residents displayed a notably higher probability of reimbursement compared to their urban counterparts (*p* = 0.002). However, for UEBMI enrollees, the reimbursement ratio was significantly higher among urban residents than their rural counterparts (*p* = 0.012). Moreover, a positive association was observed between a higher level of education and an increased likelihood of reimbursement for UEBMI enrollees (*p* = 0.041).

Under the URRBMI scheme, employed and unemployed groups exhibited significant differences in reimbursement amounts (*p* = 0.003). Notably, when comparing income groups within the URRBMI, enrollees with lower income levels exhibited a higher likelihood of reimbursement (*p* = 0.002). These nuanced findings underscore the influence of residency, educational attainment, employment status, and income levels on the distribution of outpatient benefits within the respective insurance schemes.

### 3.3 Regression results

Utilizing a two-part model, we conducted adjustments to account for the potential confounding effects of healthcare need factors on the outpatient benefits received, and the estimation results are presented in Table 3.

**Table 3:** Regression results using two-part models.

Variables	URRBMI enrollees			UEBMI enrollees		
	Probability of getting reimbursed (*n* = 14,581)	Reimbursement amounts (*n* = 341)	Reimbursement ratio (*n* = 341)	Probability of getting reimbursed (*n* = 830)	Reimbursement Amounts (*n* = 142)	Reimbursement ratio (*n* = 142)
Gender (ref. = male)						
Female	−0.001 (0.116)	−0.236 (0.205)	0.700(3.26)	0.042 (0.267)	−0.604 (0.394)	−1.351 (6.652)
Age (ref. = 45–55)						
56–65	0.224 (0.144)	0.015 (0.249)	4.988 (3.95)	0.144 (0.331)	0.489^*^ (0.469)	10.897^*^ (7.912)
>65	0.211 (0.146)	0.119 (0.249)	4.571 (3.95)	0.039 ^*^(0.324)	−0.226 (0.454)	12.390^*^ (7.657)
Marital status (ref. = married)						
Others	−0.078 (0.163)	0.113 (0.280)	10.97^*^ (4.44)	−0.489 (0.492)	0.590 (0.744)	1.896 (12.546)
Self-rated health (ref. = very poor)						
Poor	−0.079 (0.176)	−0.655^*^ (0.301)	−0.571 (4.771)	−0.425 (0.678)	0.887 (0.912)	−2.971 (5.370)
Fair	−0.865^***^ (0.188)	−0.747^*^ (0.320)	2.489 (5.083)	−0.784 (0.631)	1.156 (0.818)	−1.984 (3.779)
Good	−1.374^***^ (0.305)	−0.658 (0.527)	−1.019 (8.373)	−1.307^*^ (0.737)	−0.256 ^*^ (0.096)	−9.462^*^ (6.784)
Very good	−1.910^***^ (0.381)	−1.014 (0.648)	−19.668^*^ (10.294)	−0.603^**^ (0.504)	0.018 (0.910)	−2.736 ^*^ (5.332)
Chronic disease status (ref. = no)						
Yes	0.247^*^ (0.117)	0.168 (0.201)	−1.486 (3.197)	0.045 (0.269)	0.431 (0.387)	−2.679 (6.527)
Physical disabilities (ref. = no)						
Yes	0.078 (0.281)	0.134 (0.487)	−3.300 (7.735)	−0.299 (1.088)	0.954 (1.521)	22.837 (15.622)
Body pain (ref. = none)						
A little	0.290(0.152)	0.108 (0.266)	−6.424 (4.225)	0.008 (0.293)	−0.313 (0.428)	2.430(7.209)
Quite	0.508^**^(0.169)	−0.061 (0.295)	−3.921 (4.691)	0.392 (0.459)	0.157 (0.604)	−11.148 (10.186)

The figure in parentheses is a Standard Error. ^*^p < 0.05; ^**^p < 0.01; ^***^p < 0.001.

Regarding the probability of receiving reimbursements, the results reveal associations with age, self-rated health, chronic disease status, and body pain. Specifically, individuals of advanced age, those with chronic diseases, and those experiencing body pain exhibited a heightened likelihood of receiving reimbursements for outpatient expenses. Conversely, individuals with better self-rated health displayed a lower probability of obtaining outpatient benefits.

Within the subset of benefit recipients, it was observed that older enrollees covered by UEBMI received significantly higher reimbursement amounts and ratios. Additionally, recipients with elevated health levels were reported to experience lower reimbursement amounts and ratios. These findings underscore the nuanced interplay between demographic and health-related factors in shaping the probability and extent of outpatient benefits under the respective insurance schemes.

### 3.4 Benefits equity of outpatient pooling scheme

**Figure 2:**
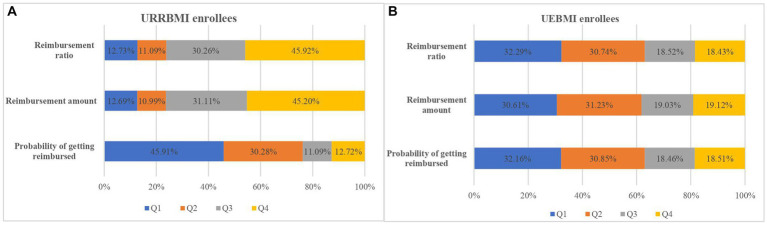
Shares of need-adjusted benefits by SES quartiles for URRBMI **(A)** and UEBMI enrollees **(B)**.

Table 4 and Figure 2 present the distribution of benefits from the outpatient pooling scheme across synthetic SES quartiles, derived through PCA and adjusted for healthcare need. The examination of benefit distribution inequality is further facilitated through the CI and Concentration curve.

**Table 4:** Shares and CIs of need-adjusted benefits by SES quartiles.

SES (quartiles)	URRBMI enrollees			UEBMI enrollees		
	Probability of getting reimbursed	Reimbursement amount	Reimbursement ratio	Probability of getting reimbursed	Reimbursement amount	Reimbursement ratio
Q1, %	45.91	12.69	12.73	32.16	30.61	32.29
Q2, %	30.28	10.99	11.09	30.85	31.23	30.74
Q3, %	11.09	31.11	30.26	18.46	19.03	18.52
Q4, %	12.72	45.20	45.92	18.51	19.12	18.43
CI (SE)	−0.0760^*^ (0.0027)	0.0708^*^ (0.0038)	0.0761^*^ (0.0026)	−0.0514^*^ (0.0124)	−0.0693^*^ (0.0179)	−0.0502^*^ (0.0122)

^*^p < 0.001.

**Figure 3:**
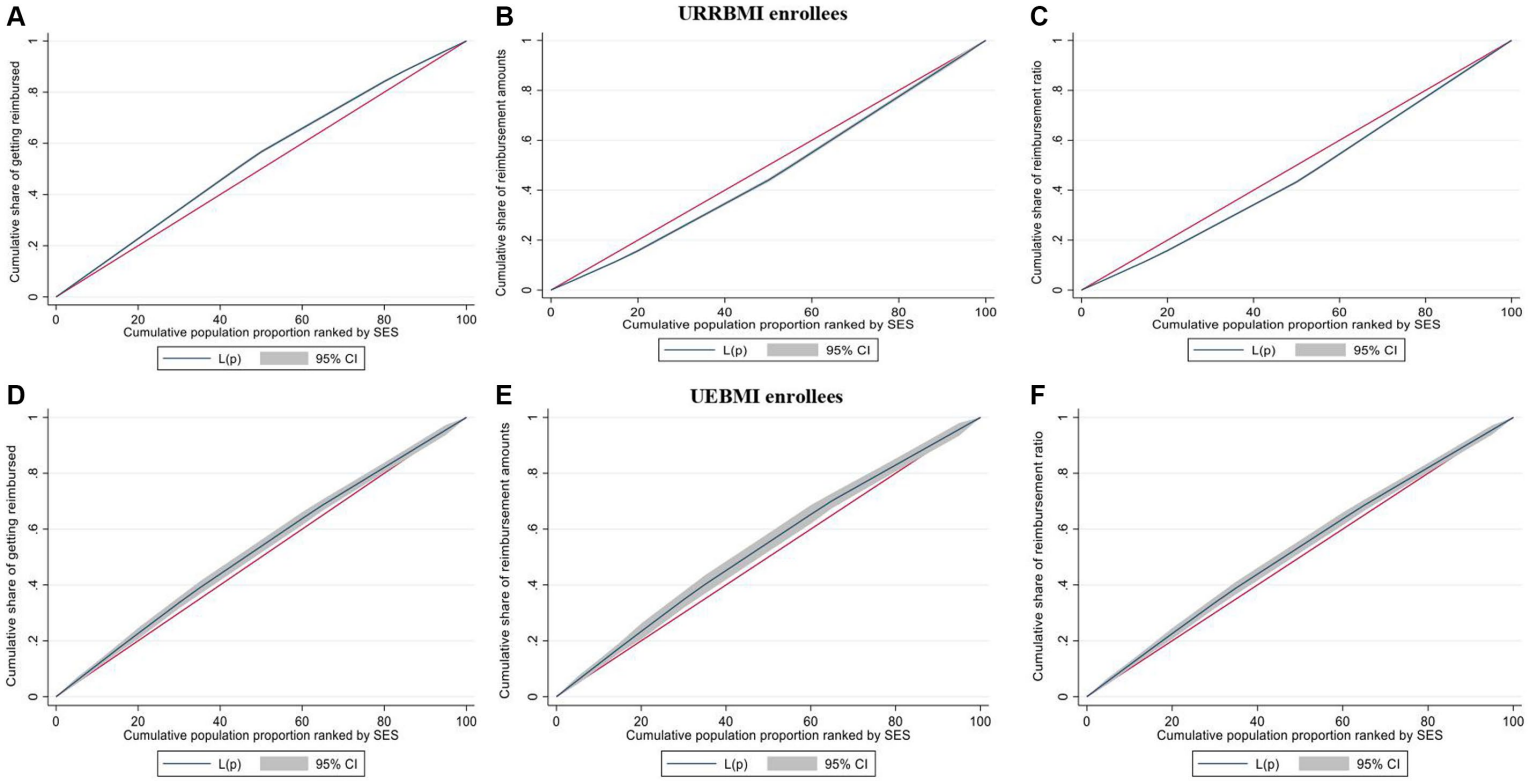
Concentration curves of probability of getting reimbursed, reimbursement amounts and reimbursement ratio for URRBMI enrollees **(A–C)** and UEBMI enrollees **(D–F)**.

Among beneficiaries, it is noteworthy that 42.44% of URRBMI enrollees and 32.16% of UEBMI enrollees belonged to the poorest SES quartiles, while the richest SES quartile accounted for 12.72 and 18.51%, respectively (Figure 2). The CI values for the probability of receiving reimbursements were − 0.0760 for URRBMI enrollees and − 0.0514 for UEBMI enrollees, and the corresponding concentration curves consistently appeared above the 45-degree line (Figure 3). These outcomes suggest the presence of pro-poor inequality in the likelihood of receiving reimbursements under the outpatient pooling scheme.

In terms of the degree of outpatient benefits among URRBMI enrollees, the highest SES quartile received 45.20% of the total reimbursed amounts, while the lowest SES quartile received merely 12.69% (Figure 2). Similar disparities were observed in reimbursement ratios. The CI values for reimbursement amounts and ratios under the URRBMI program were 0.0708 and 0.0761, respectively, with the corresponding concentration curves consistently below the 45-degree line (Figure 3). Consequently, a pro-rich inequality in the degree of benefits became evident among URRBMI enrollees post-implementation of the outpatient pooling scheme.

Contrastingly, for UEBMI enrollees, both the reimbursement amount and ratio favored the lower SES group. The lowest SES quartile captured 30.61% of the reimbursement amount and 32.29% of the reimbursement ratio, while the highest SES quartile received 19.12 and 18.43%, respectively. All CI values were negative and statistically significant, signifying that economically disadvantaged UEBMI enrollees derived more benefits under the outpatient pooling scheme.

## 4 Discussion

To our best knowledge, this is the first study to evaluate the equity in the distribution of outpatient benefits among URRBMI and UEBMI enrollees under the outpatient pooling scheme in China. Aligned with the principle of “equal treatment for equal needs,” wherein individuals with comparable healthcare needs should receive commensurate benefits, our analysis, following adjustments for the impact of healthcare need factors, unearthed two pivotal findings. Firstly, a pro-poor distribution pattern emerged in the probability of receiving benefits under the outpatient pooling scheme. This underscores a positive alignment with principles of equity, suggesting that those with socioeconomically disadvantaged backgrounds have an increased likelihood of benefiting from the scheme. Secondly, a noteworthy disparity in the distribution of benefits surfaced in terms of degree. Specifically, among URRBMI enrollees, the shift favored higher socioeconomic status (SES) groups, indicating a pro-rich trend. In contrast, economically disadvantaged UEBMI recipients experienced a more favorable distribution, receiving increased reimbursement amounts and ratios. These findings shed light on the nuanced dynamics of benefit distribution under the outpatient pooling scheme, emphasizing the importance of socioeconomic considerations in shaping the outcomes of health insurance programs in China.

Empirical evidence suggests that the disparate distribution of insurance benefits among SES groups stems from inequities in healthcare utilization (11, 32). Prior to the implementation of the outpatient pooling scheme in China, compensation for outpatient expenses was severely constrained (33, 34). This resulted in elevated OOP payments for outpatient services, particularly impacting low-income groups. Existing research has consistently demonstrated that individuals with lower economic means are more responsive to healthcare service prices (19, 35). With the introduction of the outpatient pooling scheme, there has been a notable reduction in cost-sharing for outpatient expenses. This strategic shift aims to alleviate the financial burden on individuals seeking outpatient care. Consequently, lower SES groups are incentivized to address their healthcare needs and utilize outpatient services more effectively. This change in dynamics contributes to the observed higher probability of obtaining reimbursements among individuals from lower SES backgrounds. The outpatient pooling scheme, by mitigating financial barriers, serves as a catalyst in fostering equitable access to outpatient care among different socioeconomic strata.

However, it is alarming to see that socioeconomic advantaged recipients receive more benefits from the outpatient pooling scheme under URRBMI. One potential explanation for this phenomenon lies in the inadequacy of compensation for outpatient expenses within the current URRBMI outpatient pooling scheme. Owing to limited pooled funds, URRBMI prioritizes its financial benefits toward inpatient care, considering it a major contributor to preventing households from experiencing catastrophic health expenditures (36). Although the outpatient pooling scheme was introduced, the reimbursement ratio across most of China’s prefectures is capped at not exceeding 50%, and the reimbursement ceiling is relatively modest (22, 37). The introduction of a compensation mechanism for outpatient expenses could assist low-income enrollees in overcoming budgetary constraints to access healthcare. However, the extent of access is contingent on the intricacies of the payment system (38).

Prior research has indicated that wealthier individuals are more inclined to utilize expensive specialist services, while the lower pricing of general practitioner services is more attractive to those with lower economic means (39). Moreover, the uneven distribution of medical resources in China, with high-quality healthcare services predominantly concentrated in large cities and central districts, exacerbates the barriers faced by economically disadvantaged individuals in accessing a broader spectrum of outpatient services (40). Insights from other countries also underscore that high coinsurance rates may not significantly enhance service utilization among the economically disadvantaged (19). Consequently, individuals with lower socioeconomic status are less likely to receive equivalent reimbursement amounts as their more affluent counterparts, thereby perpetuating inequities.

In addition to the design of financial benefits, various social factors contribute to the observed pro-rich inequality. Drawing upon the fundamental cause theory, individuals with higher socioeconomic status (SES) possess greater access to health-promoting resources (41, 42). It is plausible to infer that individuals with higher educational attainment are more adept at leveraging their advantages to access and comprehend information regarding the outpatient pooling scheme, thereby maximizing their benefits. Furthermore, given the substantial urban–rural disparity in the distribution of healthcare resources in China, urban enrollees experience enhanced accessibility to outpatient care compared to their rural counterparts (43). Consequently, socioeconomically advantaged groups are positioned to enjoy a larger share of insurance benefits by leveraging their ability to access a greater volume of outpatient care. This multifaceted interplay of social factors underscores the complex nature of pro-rich inequality in the distribution of insurance benefits within the context of the outpatient pooling scheme.

An additional encouraging trend observed is the pro-poor distribution of benefit degree among UEBMI recipients. Unlike the fixed-premium schemes for URRBMI, UEBMI operates on a wage-based financing model in China. UEBMI stands out as the largest social medical insurance plan in China, boasting substantial fund revenue and surplus. Data reveals that the annual contribution *per capita* for UEBMI and URRBMI in 2020 amounted to 4,566 yuan and 896 yuan, respectively (44). This discrepancy in contributions inherently translates into disparities in benefits. Given the significantly larger pooled fund under UEBMI, coupled with its provision of more robust compensation for outpatient expenses under the outpatient pooling scheme (45), it’s evident that UEBMI enrollees reap greater benefits.

Aligned with the principle of higher price elasticity of healthcare demand among lower socioeconomic status (SES) groups, economically disadvantaged UEBMI enrollees are incentivized to seek increased access to outpatient services (46, 47). Consequently, the reimbursement amounts they obtain also witness an upward trajectory. A study conducted in Taiwan similarly corroborated the notion that wage-based premium schemes can substantially enhance vertical benefits equity (26).

Furthermore, UEBMI enrollees predominantly comprise the regular wage-earning population in China. Unlike URRBMI enrollees, this demographic segment typically exhibits less variation in terms of income, residency, and employment status. Considering the positive association between SES and access to health services (48), it is reasonable to infer that the utilization of outpatient care and insurance benefits is proportionately distributed among UEBMI enrollees.

To ensure the equitable distribution of benefits, prioritizing further reforms to the outpatient pooling scheme in China is imperative. Despite commendable efforts within China’s basic medical insurance system to enhance the reimbursement ratio and broaden the scope of coverage, economic barriers to accessing outpatient care persist, particularly for disadvantaged Urban and Rural Resident Basic Medical Insurance (URRBMI) enrollees (49, 50). It is highly recommended to augment outpatient financial benefits within the URRBMI scheme, coupled with the consideration of distinct reimbursement standards tailored to vulnerable groups, such as older adults and chronic disease patients. Additionally, addressing the urban–rural disparity in the distribution of health resources and enhancing the accessibility of outpatient care for socioeconomically disadvantaged populations must be underscored as pivotal objectives. Such concerted efforts are essential to realize the overarching goal of equitable distribution of benefits within China’s healthcare landscape.

Several limitations in this study warrant acknowledgment. Firstly, the self-reported nature of outpatient expenses and reimbursement amounts introduces the potential for recall bias. Secondly, the absence of data on the severity of enrollees’ illnesses limits the comprehensive assessment of their health conditions, despite adjustments made for healthcare needs. Thirdly, the omission of an analysis on outpatient benefits received at different levels of facilities may result in an incomplete understanding of benefits distribution, given the acknowledged disparity in the quality of health services across various facility tiers in China. This limitation arises from the unavailability of relevant information in the China Health and Retirement Longitudinal Study (CHARLS). Fourthly, the age range covered in our study is limited to individuals aged 45 and above. Expanding the scope to include all age groups would provide a more holistic perspective. Fifthly, although the sample size is adequate for analyzing the distribution of benefit degree, the proportion of URRBMI enrollees receiving reimbursement is notably small. The potential increase in the number of enrollees enjoying outpatient benefits, driven by improvements in insurance generosity in China, could impact the generalizability of our findings. Continuous monitoring of the distribution of outpatient benefits is essential to ensure the ongoing relevance and applicability of our study results.

## 5 Conclusion

The implementation of an outpatient pooling scheme has augmented the likelihood of disadvantaged enrollees receiving benefits in China. However, despite this progress, pro-rich inequality persists among URRBMI recipients in terms of benefit degree. Nevertheless, there is a silver lining as it appears that economically disadvantaged UEBMI enrollees derive greater benefits from the outpatient pooling scheme. Expanding outpatient financial benefits, adopting distinct reimbursement standards and improving the accessibility of outpatient care for disadvantaged groups are highly recommended to achieve the insurance benefits equity in China and other developing countries.

## Data availability statement

The original contributions presented in the study are included in the article/Supplementary materials, further inquiries can be directed to the corresponding author.

## Ethics statement

The studies involving humans were approved by all participants provided written informed consent, and survey protocols were approved by the Peking University Ethics Review Board (IRB00001052-11015). The studies were conducted in accordance with the local legislation and institutional requirements. The participants provided their written informed consent to participate in this study.

## Author contributions

TZ: Writing – review & editing, Writing – original draft, Software, Conceptualization. MC: Writing – review & editing, Writing – original draft, Supervision.
