# Supplementary material for: Inequality in benefit distribution of reducing the outpatient cost-sharing: evidence from the outpatient pooling scheme in China
Source: Front Public Health. 2024 Mar 4;12:1357114. doi: 10.3389/fpubh.2024.1357114 (PMC10945005; doi:10.3389/fpubh.2024.1357114)
Supplement: Supplementary file 1 [file Table_1.DOCX]

**Supplementary file 1**

**Table S1 Comparison of policies for UEBMI and URRBMI enrollees.**

|  | UEBMI | URRBMI |
| --- | --- | --- |
| Target population | Urban formal employees | Residents with rural or urban hukou |
| Percentage of the total population insured in 2021 | 25.9% | 74.1% |
| Initiation time | 1997 | 2016 |
| Principle of participation | Mandatory | Voluntary at the household level |
| Government premium subsidy | None | 67% of total on average |
| Employer premium contribution | 75% of total on average | No |
| Individual premium contribution | 25% of total on average | 33% of total on average |
| Premium-paid base | Relevant to employee’s wage | None |
| Designated health facilities | All levels of public health facilities | All levels of public health facilities |
| Covered service | Inpatient services, catastrophic outpatient services, and some prevention care services | Inpatient services and catastrophic outpatient services |
| Reimbursement | Prefectural governments set deductibles, reimbursement rates, and ceilings; reimbursement rates depend on the types of health providers | Prefectural governments set different deductibles, reimbursement rates, and ceilings; reimbursement rates depend on the types of health providers |

**Table S2 Prefectures with and without implementing outpatient pooling scheme under the UEBMI in 2018**

| **Implemented outpatient pooling** | **Unimplemented outpatient pooling** |
| --- | --- |
| Beijing, Shanghai, Ningbo, Zhangzhou, Fuzhou, Guangzhou, Taizhou, Huzhou, Foshan, Jiangmen, Maoming, Qingyuan, Shenzhen, Suzhou, Lianyungang, Qingdao, Tianjin, Taizhou, Hangzhou, Yancheng, Anshan, Huhehaote, Lishui, Shijiazhuang, Xuzhou, Jiamusi, Jingmen, Dalian, Jinan, Weifang, Binzhou | Akesu, Anqing, Anyang, Baoding, Baoji, Baoshan, Benxi, Bozhou, Cangzhou, Changde, Changsha, Chaohu, Chaoyang, Chaozhou, Chengde, Chengdu, Chifeng, Chongqing, Chuxiong, Dezhou, Dingxi, Enshi, Fuyang, Ganzhou, Ganzi, Guangan, Guangzhou, Guilin, Haidong, Hanzhong, Harbin, Hechi, Hinggan, Huainan, Huanggang, Hulunbuir, Jian, Jiaozuo, Jiaxing, Jilin, Jingdezhen, Jinzhou, Jiujiang, Jixi, Kunming, Lanzhou, Liangshan, Liaocheng, Lijiang, Lincang, Linfen, Linyi, Liuan, Loudi, Luoyang, Meishan, Mianyang, Nanchang, Nanchong, Nanning, Neijiang, Ningde, Pingdingshan, Pingliang, Putian, Puyang, Qiandongnan, Qiannan, Qiqihar, Shangrao, Shaoyang, Siping, Suqian, Weihai, Weinan, Xiangfan, Xilingol, Xinyang, Xinzhou, Yangquan, Yangzhou, Yibin, Yichun, Yiyang, Yueyang, Yulin, Yuncheng, Zaozhuang, Zhangye, Zhaotong, Zhengzhou, Zhoukou, Ziyang |

**Table S3 Variables and measurements**

| **Variables** | **Measurement** |
| --- | --- |
| *benefit outcomes* |  |
| Probability of receiving reimbursements | A binary variable indicating whether an enrollee get benefits in the past month; 1=yes ,0=no |
| Reimbursement received | A continuous variable (measured by Renminbi) indicating the absolute amount of received reimbursement for outpatient expenses in the past month. |
| Reimbursement ratio | A continuous variable (measured by percentage) indicating the ratio of obtained reimbursements to the total outpatient expenses. |
| *Health-care need variables* |  |
| Gender | A binary variable scored as 1 for male and 2 for female |
| Age | A categorical variable indicating a respondent´s age; 1=‘45-55’, 2=‘56-65’, 3=‘＞65’ |
| Marital status | A binary variable indicating a respondent´s marital status; 1=married, 2=others |
| Self-rated health | A categorical variable to reflect the self-rated health status of respondents; 1=very poor, 2=poor, 3=fair, 4=good, 5=very good. |
| Chronic disease status | A binary variable indicating whether a respondent is diagnosed with chronic disease (hypertension, diabetes, stroke, myocardial infarction, tumour, asthma, fracture); 1=yes ,0=no |
| Physical disabilities | A binary variable indicating whether a respondent suffers from physical disabilities; 1=yes, 0=no |
| Body pain | A categorical variable to reflect the degree of body pains of respondents; 1=none, 2=a little, 3=quite. |
| *Socio-economic status* |  |
| Residency | A binary variable indicating residence area of respondents; 1=urban, 2=rural |
| Educational level | A categorical variable indicating a respondent´s highest level of education; 1= illiteracy, 2=primary school, 3= middle school, 4= college or above |
| Employment status | A binary variable indicating whether a respondent was employed; 1=employed ,0=unemployed |
| Individual annul income | A categorical variable indicating individual annul income; 1= ‘<3000 yuan’, 2= ‘3000-6000 yuan’, 3= ‘6001-10000 yuan’, 4= ‘＞10000 yuan’ |

**Table S4 Eigenvalues and corresponding contributions**

| Principal component | URRBMI enrollees | | | UEBMI enrollees | | |
| --- | --- | --- | --- | --- | --- | --- |
|  | Eigenvalue | Contribution (%) | Accumulate contribution (%) | Eigenvalue | Contribution (%) | Accumulate contribution (%) |
| 1 | 1.401 | 35.036 | 35.036 | 1.462 | 36.542 | 36.542 |
| 2 | 1.172 | 29.305 | 64.341 | 1.054 | 26.358 | 62.900 |
| 3 | 0.780 | 19.493 | 83.834 | 0.963 | 24.066 | 86.967 |
| 4 | 0.647 | 16.166 | 100.00 | 0.521 | 13.033 | 100.00 |

**Table S5 Principal component coefficients (loadings)**

|  | URRBMI enrollees | | UEBMI enrollees | |
| --- | --- | --- | --- | --- |
|  | 1st loading | 2nd loading | 1st loading | 2nd loading |
| Residency | -0.593 | 0.601 | 0.854 | 0.017 |
| Educational level | 0.696 | 0.167 | -0.479 | 0.755 |
| Employment status | 0.144 | 0.871 | 0.633 | 0.660 |
| Individual annul income | 0.738 | 0.155 | -0.321 | 0.219 |
